# Mechanical Performance of Novel 3D-Printed Symmetric Corrugated Hierarchical Honeycombs

**DOI:** 10.3390/polym18101233

**Published:** 2026-05-18

**Authors:** Derui Zhang, Junpeng Ma, Long Liu, Yan Zhu, Anfu Guo, Peng Qu, Shuai Guo, Zengrui Song, Yaqin Song, Shaoqing Wang

**Affiliations:** 1 School of Mechanical and Automotive Engineering, Liaocheng University, Liaocheng 252000, China; 13561461075@163.com (D.Z.); 18864988762@163.com (J.M.); guoanfu@lcu.edu.cn (A.G.); qupeng@lcu.edu.cn (P.Q.); songzengrui@lcu.edu.cn (Z.S.); 2 Shaanxi Special Equipment Inspection and Testing Institute, Xi’an 710048, China; nhyzll@163.com; 3 School of Materials Science and Engineering, Liaocheng University, Liaocheng 252000, China; shuaiguo@lcu.edu.cn; 4 State Key Laboratory for Strength and Vibration of Mechanical Structures, School of Aerospace Engineering, Xi’an Jiaotong University, Xi’an 710049, China; yqsong@mail.xjtu.edu.cn

**Keywords:** hierarchical honeycombs, friction coefficient, compressive properties, finite element analysis, energy absorption

## Abstract

Symmetric corrugated hierarchical honeycombs (SCHHs) are critical lightweight load-bearing structures, featuring distinctive topological architectures and excellent mechanical performance. However, they are prone to local buckling under out-of-plane compression and shear loading, which degrades their overall load-bearing capacity. To address this limitation, this work proposes an innovative dual-optimization strategy integrating cylindrical support structure introduction and nano-silica (SiO_2_) matrix modification to synergistically enhance the compressive and tribological properties of SCHHs. 3D-printed SCHHs and their reinforced variant (SCHH-AC) with embedded cylindrical supports were fabricated, and the effects of nano-SiO_2_ modification (0–9 wt.%) on the compressive performance and tribological behavior of the photopolymer resin matrix were systematically investigated. Experimental results demonstrate that the SCHH-AC-7% SiO_2_ configuration achieves optimal compressive performance. A critical SiO_2_ concentration threshold was identified: agglomeration at 9 wt.% induces severe mechanical degradation. Tribological tests confirm that SiO_2_ incorporation effectively reduces the resin matrix’s friction coefficient and wear rate, with the 7 wt.% concentration yielding the lowest wear rate. Additionally, geometric parametric analysis reveals that increasing the corrugation period number and amplitude further enhances SCHH’s compressive strength and energy absorption. This study establishes a theoretical and experimental foundation for the structural design and material modification of lightweight honeycombs, advancing their practical application in high-performance engineering fields demanding lightweight load-bearing and wear resistance.

## 1. Introduction

Lightweight structures are widely applied in lightweight components for aerospace, transportation, architectural engineering, and protective equipment fields due to their low density and high specific strength and specific stiffness, as well as excellent impact resistance and energy absorption characteristics [1,2,3,4,5,6,7]. Porous cellular structures represent an important branch of lightweight structures, among which honeycomb structures stand as the most typical and widely utilized configuration. They have attracted extensive attention due to their unique topological features and outstanding mechanical advantages [8,9,10]. With the continuous upgrading of equipment in various fields toward high load-bearing capacity, multi-functionality, and wear resistance, increasingly stringent comprehensive requirements have been proposed for the compressive mechanical properties, energy absorption characteristics, and surface friction and wear performance of honeycomb structures.

In recent decades, researchers have carried out extensive studies on the performance improvement of honeycomb structures, covering structural configuration design, composite material modification, and structural optimization. In terms of structural design, numerous configurations have been proposed by scholars, including vertically arranged honeycombs [11], nonlinearly graded honeycombs [12], arc-star honeycombs [13], and hierarchical honeycombs [14]. Among them, corrugated hierarchical honeycombs have become a research hotspot for lightweight load-bearing structures due to their combination of the deformation compatibility of corrugated structures and the load-bearing stability of hierarchical structures. For instance, Xu et al. [15] designed and fabricated laminated honeycomb structures with diaphragms and investigated the quasi-static and dynamic crushing responses as well as energy absorption characteristics of six bio-inspired honeycombs. The results indicated that the design of corrugated walls could effectively reduce the initial peak force of integrated honeycombs. Lin et al. [16] proposed a novel symmetric-corrugate hierarchical design strategy and described the effects of different honeycomb periods and amplitudes on deformation modes. Bai et al. [17] proposed carbon fiber-reinforced polymer (CFRP) hierarchical sandwich cylinders (HSCs) that integrate two zero Poisson’s ratio honeycomb sandwich face sheets with a corrugated core and subsequently investigated their free vibration and axial compression behaviors.

From the perspective of material modification, the incorporation of inorganic nanoparticles into polymer matrices is a well-established strategy for enhancing mechanical strength and wear resistance. For instance, Xing et al. [18] investigated the influence of monodispersed submicron spherical silica particles on the tribological performance of epoxy-based composites. Their findings revealed that the addition of a low content (0.5–4.0 wt.%) of silica fillers (with diameters of 120 nm and 510 nm) significantly reduced the wear rate of the composites. Similarly, Koh et al. [19] reported that epoxy resin filled with 33 µm SiO_2_ exhibited the minimum wear rate, whereas the unfilled epoxy resin showed the highest susceptibility to wear. In another study, Jaffer et al. [20] examined the synergistic effects of varying weight percentages of SiO_2_ particles and glass fibers, demonstrating that hardness and overall mechanical performance improved with increasing filler content. Owing to its high hardness and excellent matrix compatibility, SiO_2_ powder is extensively utilized as a reinforcing phase. While an optimal dosage of SiO_2_ can markedly enhance the elastic modulus and wear resistance of the matrix, the quantitative relationship between filler concentration and composite performance remains a subject that requires further investigation, particularly when integrated with specific structural designs [21,22,23].

Complementing material modification, integrating material modification with structural optimization is another effective approach to enhance axial load-bearing capacity [24]. Specifically, altering the cell shape of honeycomb structures can further improve their mechanical properties. However, the geometric complexity of such components poses significant challenges to traditional manufacturing methods, which struggle to achieve the required precision and may induce shape deviations that compromise the overall structural performance. In recent years, 3D printing has emerged as the preferred fabrication method for lattice sandwich structures with complex geometries, tailored spatial distributions, and specific performance requirements. According to the ISO/ASTM standards, seven mainstream additive manufacturing technologies for the fabrication of porous structures include fused deposition modeling (FDM), vat photopolymerization (VPP/DLP/SLA), selective laser sintering (SLS), selective laser melting (SLM), electron beam melting (EBM), binder jetting (BJ), and laminated object manufacturing (LOM). These processes differ significantly in material compatibility, forming accuracy, densification level, and mechanical properties, which directly affect the mechanical behaviors and failure modes of cellular structures.

Among these technologies, fused deposition modeling (FDM) has been widely employed for fabricating polymeric porous cellular structures due to its low cost and strong material compatibility. Ergene and Yalçın [25] fabricated various periodic cellular structures via FDM and found that their mechanical properties exhibited significant anisotropy, and the compressive strength and stiffness decreased approximately linearly with the increase in porosity. In contrast, vat photopolymerization technologies represented by digital light processing (DLP) have become the preferred choice for high-precision honeycomb and porous structures owing to their ultra-high forming accuracy and excellent surface quality. Zhang et al. [26] prepared ZrO_2_(3Y)/Al_2_O_3_ ceramic hollow lattice structures using photopolymerization-based 3D printing, achieving a compressive strength of 326 MPa at 35% porosity. This result verified that such processes enable the integrated fabrication of lightweight porous ceramic components with high precision and superior strength.

Numerous studies have confirmed the feasibility of 3D printing for honeycomb structure fabrication: Mirzaei et al. [27] investigated the mechanical properties of composite sandwich structures with 3D-printed honeycomb cores, highlighting that 3D-printed structures offer customizable geometries and controlled material distribution, thereby enhancing material strength and stiffness. Li et al. [28] proposed a novel standing surface acoustic wave (SSAW)-assisted digital light processing (DLP) 3D printing method, developed an acoustically assisted printing system, and verified its capability to fabricate fiber-reinforced honeycomb structures. Habib et al. [29] studied the in-plane static compression crushing behavior and energy absorption capacity of 3D-printed polymeric honeycomb structures with different cell thicknesses, confirming that 3D printing can produce honeycomb structures with tailored geometries, adjustable energy absorption capacities, and predictable performance. Notably, photopolymer resin 3D printing enables the precise fabrication of micro-scale honeycomb structures [27,30,31,32,33,34], providing process support for the collaborative optimization of structure and material.

To the best of the authors’ knowledge, extensive research has been conducted on honeycomb structure optimization. However, pure resin-based symmetric corrugated hierarchical honeycombs (SCHHs) suffer from two critical performance shortcomings: low surface hardness, which renders them prone to surface wear under actual friction and wear conditions, accompanied by a high friction coefficient and large wear rate that fail to meet long-term wear-resistant service requirements; and susceptibility to local buckling under out-of-plane compression and shear loading, leading to degraded overall load-bearing capacity.

To address these dual challenges, this study focuses on SCHHs and implements multidimensional performance optimization through the integration of structural innovation and material modification; its primary innovation lies in mimicking the continuous support of vascular bundles in plant stem vascular cambium [35,36] by introducing corrugation-inscribed cylindrical structures inside honeycomb cells, which is expected to resolve the insufficient axial support of SCHH. Pure resin and SiO_2_-modified resin-based honeycomb specimens were fabricated via 3D printing technology. Specifically, we systematically investigated the effects of cylindrical structure introduction and SiO_2_ concentration on the compressive performance of SCHH-AC, clarified the mechanism by which high-concentration SiO_2_ agglomeration degrades structural performance, explored the regulatory role of SiO_2_ concentration on the friction coefficient and wear rate of resin-based materials, and elucidated the correlation between corrugated geometric parameters (period, amplitude) and SCHH compressive properties.

## 2. Materials and Methods

### 2.1. Model Design

**Figure 1:**
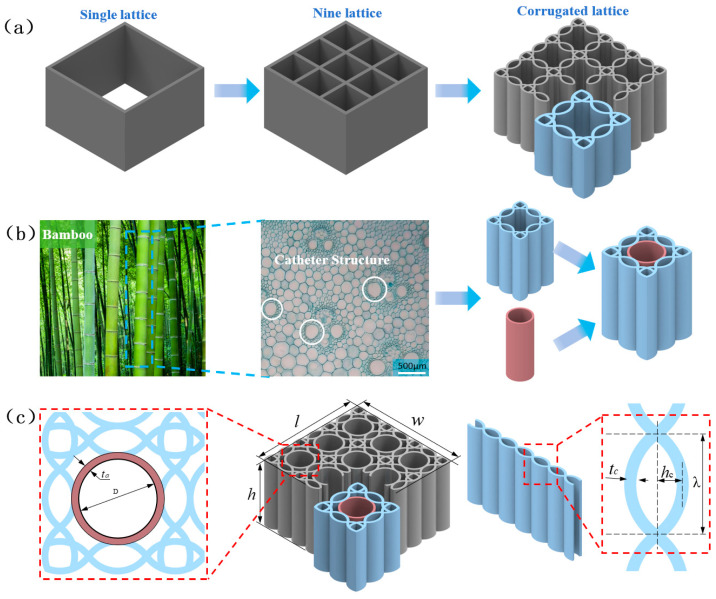
(**a**) Lattice evolution of SCHH; (**b**) cylindrical structure inspired by vascular bundle conduits in bamboo, Arrow indicates the evolution process, and the two colors represent two different structures; (**c**) schematic diagram and geometric parameters of SCHH AC.

According to previous research [37], a conventional quadrilateral honeycomb unit can be evolved into a symmetric corrugated hierarchical honeycomb (SCHH) by introducing sinusoidal corrugations and a symmetric hierarchical design, as illustrated in Figure 1a. Such structural modification effectively improves the compressive performance of honeycombs. Nevertheless, adjacent corrugated layers only provide mutual lateral support, making the structure susceptible to severe stress concentration under external loads, which inevitably deteriorates its overall load-bearing performance. Therefore, based on the above structure and inspired by the vascular bundle conduit structure of bamboo stems, this study proposes a novel bio-inspired hierarchical honeycomb configuration. Three-dimensional parametric modeling of the honeycomb structure was carried out using SolidWorks 2020 software, and key geometric parameters were defined through dimension-driven modeling to ensure full consistency between the numerical model and the experimental specimens. The evolutionary process of the honeycomb unit is depicted in Figure 1b.

Figure 1c presents the hierarchical honeycomb formed by the periodic arrangement of the above honeycomb unit, which is named symmetric corrugated hierarchical honeycomb with embedded cylindrical supports (SCHH-AC). Its key geometric parameters (wall thickness $t_{c}$, total length l, total width w, and height h) are fixed at 0.53 mm, 30.3 mm, 30.3 mm, and 18 mm, respectively; other parameters are listed in Table 1. In this bio-inspired structure, the embedded cylindrical supporting members mimic the continuous arrangement of vascular bundles in biological systems, providing efficient axial stiffness, promoting reasonable stress distribution inside the hierarchical structure, and significantly enhancing the overall load-bearing capacity of the honeycomb.

### 2.2. Specimen Preparation and Material Properties

Specimen fabrication in this study consisted of three sequential stages: preparation of the photosensitive resin mixture, vat photopolymerization printing, and post-curing treatment. Taking the formulation containing 5 wt.% SiO_2_ as an example, the photosensitive resin was initially mixed with spherical SiO_2_ powder (200 nm particle size) at a mass ratio of 100:5 in a beaker. This mixture was then subjected to ultrasonic dispersion (Ultrasonic disperser JP-010 T, Shenzhen Jiemeng Cleaning Equipment Co., Ltd., Shenzhen, China) combined with mechanical stirring (Electric stirrer JJ-160 W, Changzhou Yitong Analytical Instrument Manufacturing Co., Ltd., Changzhou, China) for 30 min to ensure homogeneous dispersion of the nanoparticles. All specimens were fabricated using an EvoDentAME RH2500 dental 3D printer (UnionTech Co., Ltd., Shanghai, China), based on the principle of ultraviolet (UV) light-induced photopolymerization. The printer features a top-down optical configuration, where the resin vat containing the liquid precursor is positioned above the optical system. Guided by pre-sliced digital models, a computer-controlled UV light source projected a series of cross-sectional images layer-by-layer onto the resin surface, inducing localized solidification. A physical prototype was subsequently formed by the layer-wise lifting of the build platform. The main parameters of the 3D printer are listed in Table 2. The commercial photopolymer resin used in this work was supplied by Shenzhen Chongguang New Material Co., Ltd., Shenzhen, China. Upon completion of printing, the specimens were carefully detached from the build platform using a spatula, and the sacrificial support structures were removed with specialized tools. To eliminate uncured resin residues, the as-printed specimens were thoroughly cleaned with 95% ethanol. Finally, a post-curing process was conducted in a UV curing oven (PCU-80, UnionTech Co., Ltd., Shanghai, China) for 15 min to enhance the mechanical integrity of the structures.

**Figure 2:**
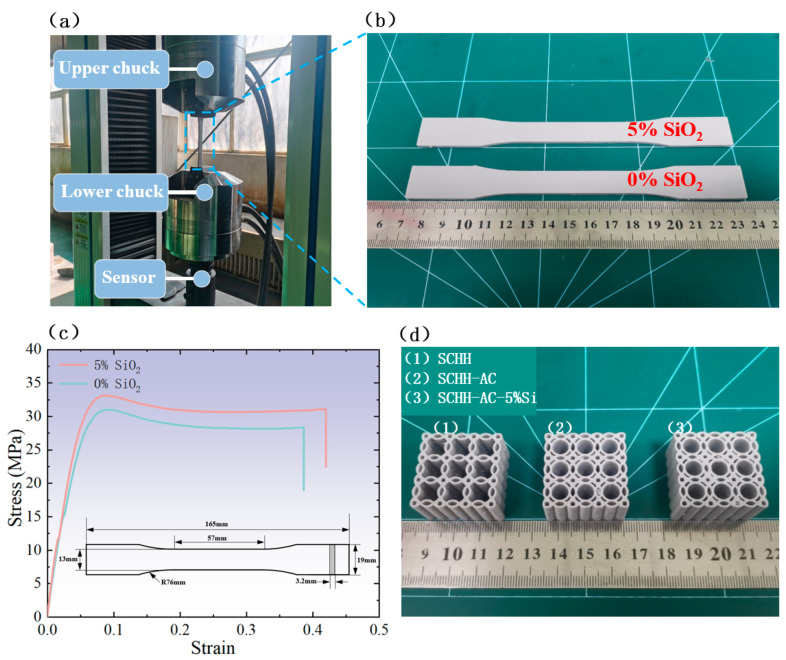
(**a**) Uniaxial tensile test module of the electronic universal testing machine; (**b**) 3D-printed standard dog-bone specimens; (**c**) stress–strain results of dog-bone specimens; (**d**) 3D-printed SCHH, SCHH-AC, and SCHH-AC-5%Si structural specimens.

To investigate the effects of SiO_2_ addition on the material parameters of the resin (including Young’s modulus and Poisson’s ratio), in accordance with ASTM D638-14 (Standard Test Method for Tensile Properties of Plastics), uniaxial tensile tests were conducted on standard dog-bone tensile specimens of resin with 5 wt.% SiO_2_ and 0 wt.% SiO_2_ using an electronic universal testing machine (Jinan Xinguang Testing Machine Manufacturing Co., Ltd., Jinan, China), as shown in Figure 2a,b. The tests were performed at a constant speed of 2 mm/min. This rate falls within the recommended range (1–5 mm/min) specified in ASTM D638-14 for rigid thermosetting resins and photocurable polymers, ensuring stable and reliable measurements of parameters such as Young’s modulus and Poisson’s ratio. The results of four parallel tests were processed to obtain the stress–strain curves illustrated in Figure 2c. In the uniaxial quasi-static tensile test, the longitudinal and transverse strains of specimens were synchronously collected by an extensometer. Within the linear elastic stage of the stress–strain curve, Poisson’s ratio was calculated as the absolute ratio of transverse strain to longitudinal strain. Young’s modulus was determined by the stress–strain ratio in the linear elastic region. The final values of both parameters were taken as the average of four parallel test results. The material properties are listed in Table 3, and the mechanical properties measured from tensile tests were adopted in the subsequent finite element simulation.

Test results show that compared with the pure resin (0 wt.%) specimens, the 5 wt.% SiO_2_-doped modified resin specimens exhibit obvious changes in elastic mechanical parameters such as Young’s modulus and Poisson’s ratio, with significantly improved fracture stress and elongation at break. The reason is that an appropriate amount of SiO_2_ nanoparticles can achieve favorable dispersion strengthening and load transfer effects in the resin matrix. The nanoparticles can effectively bear part of the applied external load, inhibit the initiation and propagation of microcracks inside the matrix, and delay the further extension and development of cracks.

### 2.3. Experimental Apparatus and Methods

**Figure 3:**
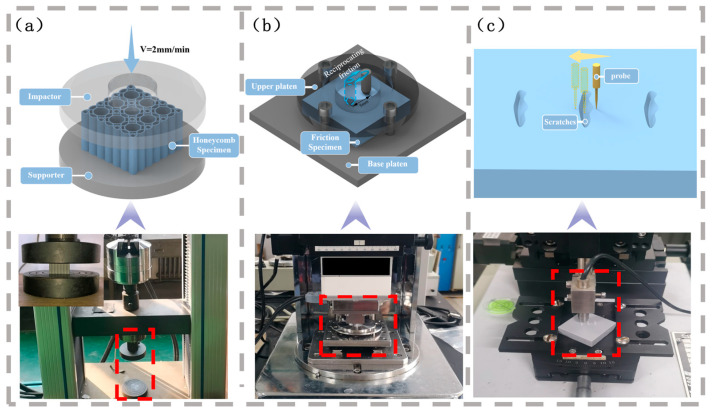
(**a**) Compression test module of the electronic universal testing machine; (**b**) CFT-I comprehensive material surface property tester; (**c**) MT-500 probe-type material surface wear scar measuring instrument.

Quasi-static compression tests were performed on all symmetric corrugated hierarchical honeycomb structures in accordance with the ISO 604:2002 standard, utilizing the compression module of an electronic universal testing machine (Jinan Xinguang Testing Machine Manufacturing Co., Ltd., Jinan, China), as schematically illustrated in Figure 3a. The test protocol was implemented in a systematic manner to ensure the reliability and reproducibility of experimental results, following the sequential steps outlined below: first, each specimen was vertically positioned at the geometric center of the upper and lower pressure plates and securely fixed to prevent lateral displacement during loading; subsequently, a uniform compressive load was applied at a constant displacement rate of 2 mm/min. Throughout the entire testing process, load and displacement data were synchronously collected and recorded to capture the complete compressive response of the structures. For each test, key mechanical parameters, including the maximum compressive load and corresponding deformation, were extracted and documented. To minimize the influence of random experimental errors and ensure statistical significance, three replicate specimens with identical material compositions and geometric specifications were fabricated and tested for each experimental group. The final experimental results presented herein are the average values of these three parallel tests.

As illustrated in Figure 3b, the friction coefficient measurements were conducted using a CFT-I comprehensive material surface property tester (Lanzhou Zhongke Kaihua Technology Development Co., Ltd., Lanzhou, China). This testing apparatus features a modular design, which enables the versatile characterization of various material surface properties, while offering a wide adjustable range of applied normal loads and sliding speeds to accommodate diverse experimental requirements. Equipped with a high-precision data acquisition system, the tester allows for the automatic and accurate measurement of key tribological parameters, including friction force and friction coefficient, under computer control, thereby eliminating manual measurement errors and ensuring data reliability. The tests were carried out in accordance with ASTM G133-22 (Standard Test Method for Linearly Reciprocating Ball on Flat Sliding Wear). A reciprocating ball-on-flat sliding mode was adopted, and all experimental parameters were strictly controlled as follows: the friction tests were performed using a reciprocating sliding mode, with the following experimental parameters strictly controlled: a normal load of 30 N, a reciprocating sliding frequency of 2 Hz (equivalent to 120 rpm), a sliding stroke of 5 mm, and a spherical indenter with a diameter of 5 mm employed as the friction counterpart. Three parallel specimens were tested under each set of experimental parameters, and each specimen was measured three times. The average value was taken as the final experimental result.

As shown in Figure 3c, the determination of wear rate was realized by an MT-500 probe-type material surface wear scar measuring instrument (Lanzhou Zhongke Kaihua Technology Development Co., Ltd., Lanzhou, China). The instrument is a device for accurately measuring the morphology of wear scars formed after friction and wear on the material surface. It can automatically measure the width and depth of wear scars, calculate the wear volume, display the two-dimensional profile of wear scars in a graphical way, and perform real-time graphical display and data storage, thus intuitively characterizing the wear status and wear resistance of the material surface. The longitudinal resolution of the scanning probe is 0.01 µm, and the longitudinal measurement range is 0–1000 µm. The data were processed by a computer-controlled automatic scanning mode, the wear scar curve was drawn, and the wear volume was automatically calculated. The wear rate can be obtained by using the relevant formula.

### 2.4. Simulation Analysis

**Figure 4:**
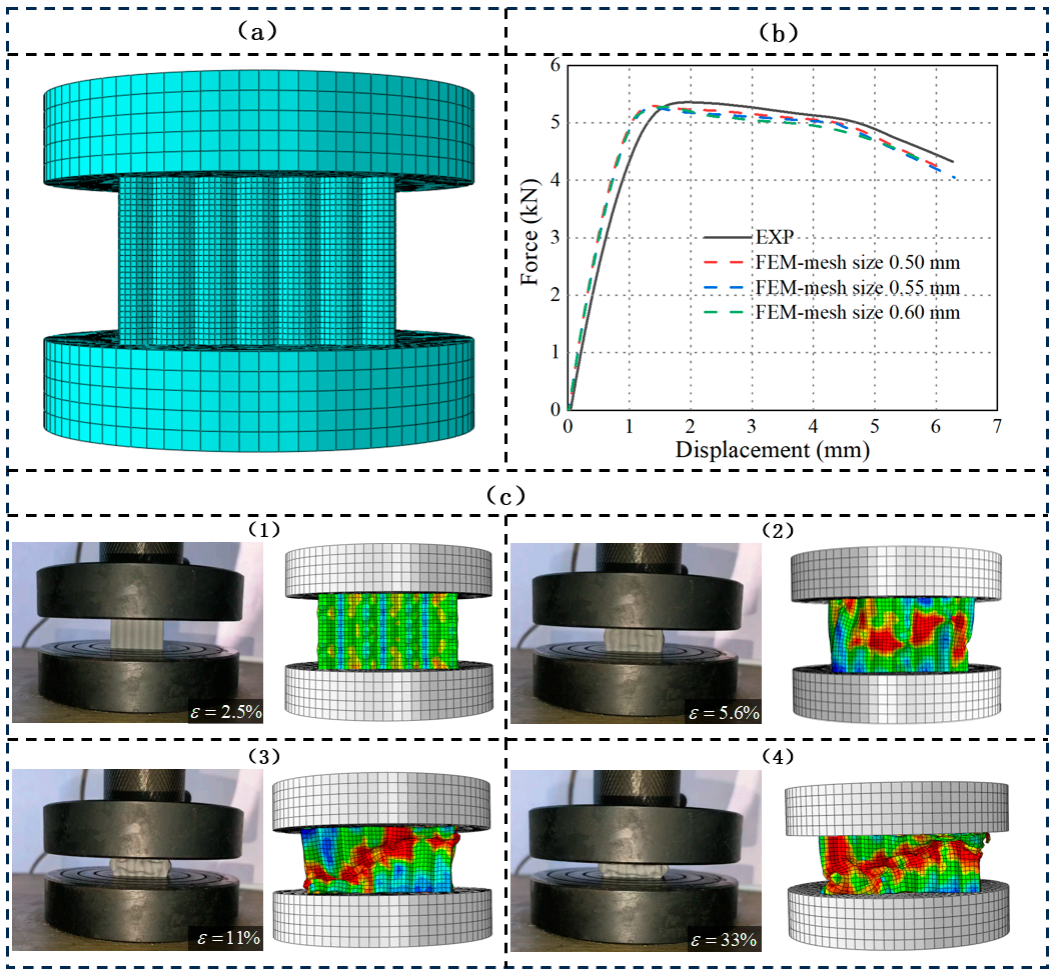
(**a**) Finite element model of the SCHH structure with a mesh size of 0.60 mm; (**b**) convergence analysis of the finite element simulation results for the SCHH structure; (**c**) comparison between experimental and simulated compressive deformations of the SCHH structure.

Based on the assumption of material isotropy, the tensile and compressive behaviors of the SCHH structure under quasi-static compression conditions are considered symmetrical. To verify the reliability of the experiment, the finite element numerical simulation of the structure was carried out using the commercial finite element analysis software ABAQUS 2019b. As shown in Figure 4a, the geometric models of two short cylinders with a diameter of 50 mm were established as the loading head and test platform, respectively. The SCHH was made of photosensitive resin material, and Q235 steel was selected for both the loading head and the test platform.

The finite element boundary conditions are set as follows: the bottom support platform is fully fixed (Fixed Constraint), with its translational degrees of freedom U1, U2, and U3 and rotational degrees of freedom UR1, UR2, and UR3 constrained. The top loading head retains only the displacement degree of freedom along the compression direction (U3), with all other translational and rotational degrees of freedom constrained. A uniform compression load is applied under quasi-static displacement control. Surface-to-surface contact is defined between the loading head and the upper surface of the SCHH, as well as between the support platform and the lower surface of the SCHH. A finite-sliding algorithm is adopted, with “hard contact” for normal behavior and frictionless contact for tangential behavior to match the experimental boundary conditions.

In finite element simulations, mesh size exerts a critical influence on both computational accuracy and efficiency: an excessively large mesh size may compromise the precision of calculation results, whereas an overly fine mesh size can lead to increased computational costs and reduced computational efficiency. To address this trade-off and ensure the reliability of subsequent simulation results, a mesh convergence analysis was performed, in which three different mesh sizes (0.50 mm, 0.55 mm, and 0.60 mm) were designed for comparative investigation; the corresponding node number and element number are listed in Table 4. As illustrated in Figure 4b, the force–displacement curves of the symmetric corrugated hierarchical honeycomb (SCHH) structure exhibited minimal deviations and high similarity when adopting the three mesh sizes (0.50 mm, 0.55 mm, and 0.60 mm). Consequently, a mesh size of 0.60 mm was selected as the optimal choice for subsequent simulations, balancing the requirements of computational accuracy and efficiency.

Furthermore, it can be observed that the force–displacement curves obtained from the quasi-static compression experiments are generally consistent with those derived from the finite element simulations, albeit with slight deviations. The primary cause of these deviations lies in the fact that the simulation model was established based on the idealized geometric dimensions of the SCHH structure, which neglects the tiny geometric defects inherently generated during the 3D printing process, including slight dimensional errors, surface roughness, and local material imperfections.

To further verify the accuracy of the finite element model in predicting the deformation behavior of the SCHH structure, as shown in Figure 4c, a comparative analysis of the deformation modes between the quasi-static compression experiment and the simulation was carried out. At different strain stages of 2.5%, 5.6%, 11%, and 33%, the simulated stress nephograms corresponded to the experimental collapse states one by one. It was found that the actual deformation morphology of the structure was almost consistent with the simulation results, which intuitively verified the reliable prediction ability of the finite element simulation for the deformation behavior.

### 2.5. Evaluation Methods

The compressive performance of various structures is generally determined by the peak load, energy absorption, and compressive modulus. Therefore, the above indicators were also used to evaluate the performance of different types of SCHH. The peak load refers to the maximum load that a component can bear under stress, and its value can be directly determined by the peak point of the load–displacement curve.

Energy absorption (*EA*) [38] refers to the mechanical energy absorbed by the material during compression, and its value is determined by the area enclosed by the load–displacement curve and the displacement axis, which is a key index to measure the energy dissipation capacity of the SCHH structure. The calculation formula of energy absorption is expressed as Equation (1):

(1) $$EA=\int_{0}^{\delta }F\left(\delta \right)d\delta$$

where EA is the energy absorption (J), representing the total energy absorbed by the material during compression; *F*(*δ*) is the compressive load as a function of displacement *δ* (kN), representing the load borne by the material at a certain displacement; and *δ* is the compressive displacement (mm), representing the deformation of the material in the compression direction.

The compression modulus [39] characterizes the stiffness of a material within the elastic deformation range, defined as the ratio of stress (*σ*) to strain (*ε*). The initial segment of the experimentally measured stress–strain curve in this study presents a nonlinear portion. This stage is mainly caused by factors such as end face fitting of the specimen, compaction of tiny gaps, and contact slippage of the test device, rather than the intrinsic elastic deformation of the material. Therefore, the initial nonlinear interval of the curve should be discarded, and the linearly elastic interval should be selected. The compression modulus *E* can be calculated by adopting Formula (2) within this approximately linear segment.

(2) $$E=\frac{\sigma }{\varepsilon }=\frac{F/A}{∆L/L_{0}}$$

where σ is the compressive stress (Pa), referring to the compressive load borne per unit area of the material; ε is the compressive strain, referring to the relative deformation of the material in the compression direction.

The wear rate ω ($10^{-4}\;{mm}^{3}/\left(N\cdot m\right)$) [40] is the wear volume generated by the material sliding one meter under per-Newton load, and a smaller value indicates better wear resistance. The relevant calculation formula is given in Equation (3):

(3) $$\omega =\frac{\nu }{F\cdot L}$$

where ν is the wear volume (mm^3^), i.e., the volume lost on the material surface due to contact and sliding, which can be measured by a probe-type material surface wear scar measuring instrument; F is the normal load (N); and *L* is the total friction travel (m), referring to the total path length covered by the friction head sliding back and forth on the specimen surface within the specified test duration.

## 3. Results

### 3.1. Comparative Study on the Compressive Performance of Symmetric Corrugated Hierarchical Honeycombs Modified by Cylindrical Support Structures and SiO_2_ Powder

To evaluate the effect of cylindrical support structures on the compressive behavior of symmetric corrugated hierarchical honeycombs (SCHHs) while keeping all other variables constant, comparative quasi-static compression experiments were conducted between the baseline SCHH (without cylindrical support structures) and the modified SCHH with cylindrical support structures (SCHH-AC). The 3D-printed specimens of SCHH and SCHH-AC are displayed in Figure 2d.

In addition, to investigate the influence of SiO_2_ powder incorporation on the compressive performance of the hierarchical honeycombs, spherical SiO_2_ powder (200 nm particle size) accounting for 5 wt.% of the total mass was mixed into the raw material of the SCHH-AC structure (photosensitive resin: SiO_2_ powder = 100:5, mass ratio), yielding a new composite structure denoted as SCHH-AC-5%Si. Comparative experiments were further performed between SCHH-AC and SCHH-AC-5%Si to assess how the addition of 200 nm spherical SiO_2_ powder further modifies the compressive behavior of the SCHH-based structures, with their 3D-printed specimens also presented in Figure 2d.

**Figure 5:**
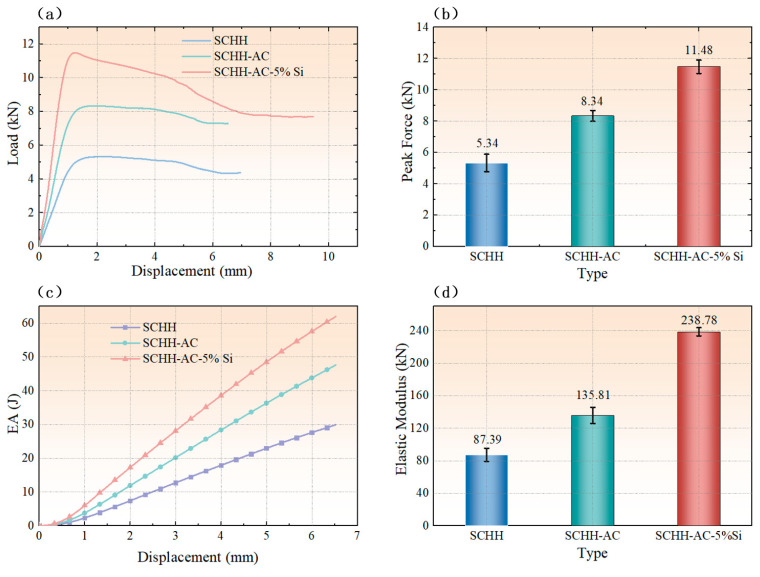
Mechanical performance comparisons among the three symmetric corrugated hierarchical honeycomb structures (SCHH, SCHH-AC, and SCHH-AC-5%Si): (**a**) load–displacement curves; (**b**) peak load comparison; (**c**) energy absorption performance comparison; (**d**) compressive modulus comparison.

Quasi-static compression tests were carried out on all three types of structural specimens (SCHH, SCHH-AC, and SCHH-AC-5%Si), and the resulting load–displacement curves are presented in Figure 5a. As illustrated in these curves and the compression process shown in Figure 4c, all three symmetric corrugated hierarchical honeycombs exhibited a consistent load–displacement response: the load increased linearly with displacement until the ultimate (peak) load was reached, followed by a gradual load decrease with further displacement until the honeycomb walls underwent buckling failure.

The peak load comparison diagram in Figure 5b clearly demonstrates that the load-bearing capacity and peak load of the three structures increased sequentially in the order of SCHH < SCHH-AC < SCHH-AC-5%Si. Specifically, the peak load of SCHH-AC was approximately 56% higher than that of the baseline SCHH, while the peak load of SCHH-AC-5%Si was about 38% higher than that of SCHH-AC. This significant improvement in compressive performance confirms that both the incorporation of cylindrical support structures and the addition of SiO_2_ powder can substantially enhance the compressive behavior of the symmetric corrugated hierarchical honeycomb.

Comparative analysis of domestic and international studies demonstrates that embedding internal supporting structures into honeycomb configurations is an effective strategy to enhance their load-bearing capacity. For example, Xia and Su et al. [41] installed columnar supporting structures inside bio-inspired honeycombs and effectively improved the impact resistance and energy absorption performance of the structure. This benefit derives mainly from the curved profile of cylindrical supports, which enables uniform load distribution and mitigates local stress concentration more effectively compared with unsupported configurations and polygonal supporting structures.

In terms of silica powder modification, extensive existing research has verified that nano-SiO_2_ can remarkably enhance the mechanical properties of resin matrix composites. Sprenger et al. [42] reported that nano-SiO_2_ significantly increases the modulus, fracture toughness, and compressive strength of epoxy resin systems. This finding is consistent with the present result that SiO_2_ powder obviously improves the peak load of SCHH-AC structures, further validating the effectiveness of nano-SiO_2_ as a reinforcing filler.

The underlying enhancement mechanisms can be elaborated as follows: the cylindrical support structures provide effective mechanical support during honeycomb deformation, enabling the specimens to better disperse external compressive forces and mitigate local stress concentrations. Meanwhile, the introduction of high-hardness SiO_2_ powder contributes to the improvement of the overall mechanical properties of the matrix material. Additionally, the energy absorption–displacement curves in Figure 5c indicate that SCHH-AC-5%Si exhibits superior energy absorption capacity compared to the other two structures. Furthermore, the compressive modulus comparison in Figure 5d reveals that SCHH-AC-5%Si possesses the highest compressive modulus among the three, further verifying the synergistic enhancement effect of cylindrical structures and SiO_2_ powder on the mechanical performance of the hierarchical honeycomb.

### 3.2. Effect of SiO_2_ Concentration on the Performance of SCHH-AC

Based on the research findings presented in Section 3.1, both the incorporation of cylindrical support structures and the addition of SiO_2_ powder were confirmed to substantially enhance the compressive performance of SCHH. Building upon this conclusion, the SCHH-AC was selected as the baseline printing configuration for the subsequent experiments. To further explore the optimal SiO_2_ concentration that maximizes the material performance, five distinct SiO_2_ concentration gradients (0%, 3%, 5%, 7%, and 9% by weight) were designed, and comparative quasi-static compression experiments were conducted accordingly.

**Figure 6:**
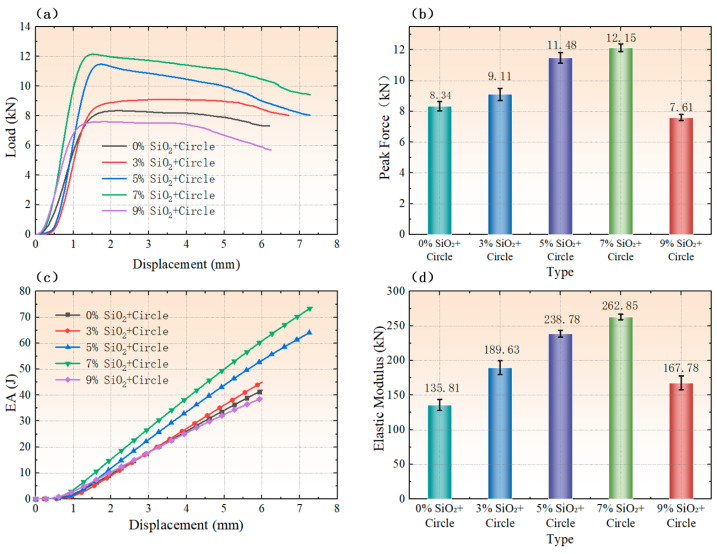
Mechanical performance comparisons of symmetric corrugated hierarchical honeycombs with cylindrical support structures (SCHH-AC) under different SiO_2_ concentration gradients: (**a**) load–displacement curves; (**b**) peak load comparison; (**c**) energy absorption performance comparison; (**d**) compressive modulus comparison.

As clearly illustrated in the peak load comparison diagram (Figure 6b), among the five concentration gradients investigated, the specimen with a 7% SiO_2_ concentration exhibited the highest load-bearing capacity. Specifically, Figure 6a,b collectively demonstrate that the load-bearing capacity and peak load of the specimens increased sequentially with increasing SiO_2_ concentration across the first four gradients (0%, 3%, 5%, and 7%). However, a sharp decline in both load-bearing capacity and peak load was observed when the SiO_2_ concentration was increased to 9%.

The energy absorption–displacement curves for different SiO_2_ concentration gradients are presented in Figure 6c. At the same displacement level, the energy absorption capacity of the specimens increased progressively with SiO_2_ concentration for the first four gradients (0%, 3%, 5%, and 7%). In contrast, the specimen with a 9% SiO_2_ concentration exhibited the lowest energy absorption capacity, even lower than that of the specimen without SiO_2_ (0% concentration).

Additionally, the compressive modulus comparison diagram (Figure 6d) reveals a similar variation trend: the compressive modulus of the specimens increased sequentially with SiO_2_ concentration across the 0%, 3%, 5%, and 7% gradients, while a drastic reduction in compressive modulus was observed when the SiO_2_ concentration reached 9%. Collectively, the results presented in Figure 6a,d indicate a consistent performance trend for the SCHH-AC across the five SiO_2_ concentration gradients: the load-bearing capacity, energy absorption capacity, and compressive modulus all increased with increasing SiO_2_ concentration up to 7%, whereas a sharp deterioration in all mechanical properties occurred when the SiO_2_ concentration exceeded this optimal value (i.e., at 9%).

**Figure 7:**
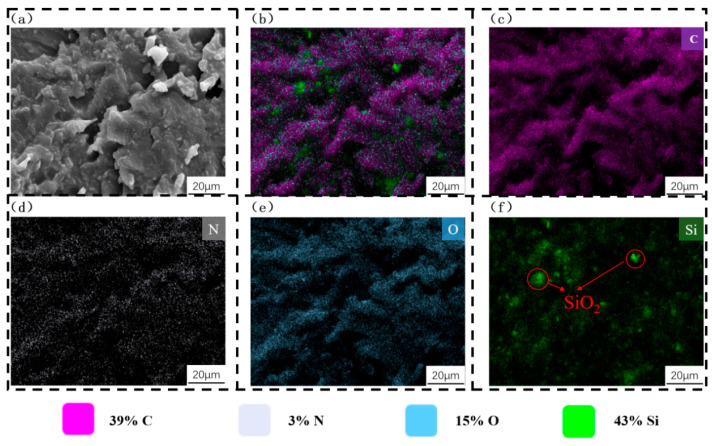
Scanning electron microscopy (SEM) micrographs and energy-dispersive X-ray spectroscopy (EDS) elemental analysis of the specimen with a 7% SiO_2_ concentration gradient: (**a**) SEM micrograph; (**b**) EDS elemental distribution map of mixed C, N, O, and Si elements; (**c**–**f**) EDS elemental distribution maps of individual C, N, O, and Si elements, respectively.

**Figure 8:**
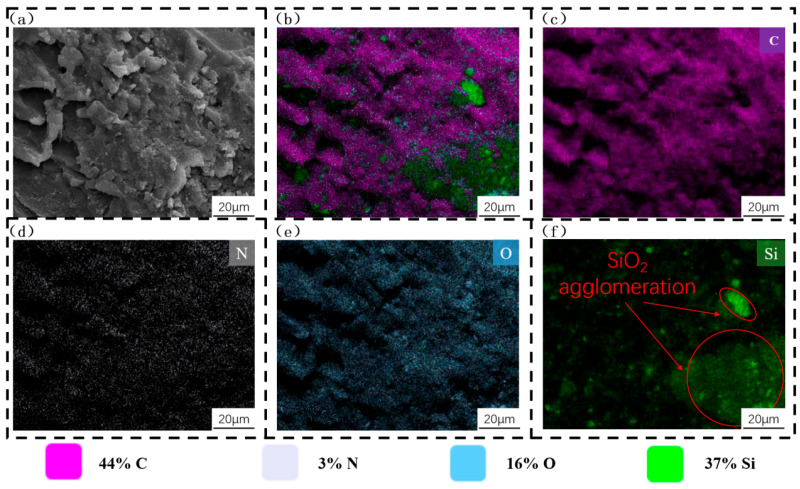
Scanning electron microscopy (SEM) micrographs and energy-dispersive X-ray spectroscopy (EDS) elemental analysis of the specimen with a 9% SiO_2_ concentration gradient: (**a**) SEM micrograph; (**b**) EDS elemental distribution map of mixed C, N, O, and Si elements; (**c**–**f**) EDS elemental distribution maps of individual C, N, O, and Si elements, respectively.

To elucidate the underlying mechanism responsible for the sharp deterioration of compressive performance at excessively high SiO_2_ concentrations, specimens with SiO_2_ concentration gradients of 7% and 9% (wt.%) were selected for scanning electron microscopy (SEM) and energy-dispersive X-ray spectroscopy (EDS) analyses. The SEM micrographs and elemental composition analyses of the specimens with 7% and 9% SiO_2_ concentrations are presented in Figure 7 and Figure 8, respectively. For the EDS analysis, carbon (C), nitrogen (N), and oxygen (O) were selected as characteristic elements to represent the organic photosensitive resin matrix, while silicon (Si) was used as the characteristic element to identify the SiO_2_ powder.

As illustrated in Figure 7, when the SiO_2_ concentration was 7%, the SiO_2_ particles were uniformly encapsulated by the organic photosensitive resin matrix, forming a tight and well-bonded interface between the two phases. Additionally, the specimen surface exhibited a homogeneous microstructure without obvious defects (e.g., cracks, voids, or agglomerations). In contrast, a distinct microstructural difference was observed when comparing Figure 7 with Figure 8: as the SiO_2_ concentration increased from 7% to 9%, the SiO_2_ particles no longer maintained uniform dispersion within the resin matrix; instead, extensive particle agglomeration occurred.

This microstructural change is consistent with the results of the quasi-static compression tests, where the specimen with 9% SiO_2_ concentration exhibited significantly lower compressive performance than that with 7% SiO_2_ concentration. Collectively, these observations lead to the conclusion that the agglomeration of SiO_2_ particles at high concentrations disrupts the original integrity of the resin matrix structure, weakens the interfacial bonding between the SiO_2_ particles and the resin, and ultimately results in a substantial reduction in the load-bearing capacity of the hierarchical honeycomb structure.

Further validation of the above mechanism can be supported by existing literature. Elmahdy et al. [43] confirmed that the uniform dispersion of nano-SiO_2_ in RTM6 epoxy resin can remarkably enhance the compressive strength and elastic modulus of composite materials. However, excessive incorporation of nano-SiO_2_ particles easily leads to uneven dispersion and local agglomeration, which induces internal stress concentration and ultimately causes a significant deterioration in the load-bearing performance of composites.

Uniyal et al. [44] indicated in their review that the uniform distribution of nano-SiO_2_ within the resin matrix is essential for achieving improved mechanical properties. Excessive addition tends to trigger particle agglomeration due to high specific surface area, thereby generating internal stress concentration sites and interfacial defects and eventually resulting in an obvious reduction in the compressive strength and load-bearing efficiency of composite materials.

Accordingly, the deterioration effect induced by particle agglomeration when the SiO_2_ content exceeds the critical value in this work is highly consistent with the conclusions in existing literature, which further verifies the general mechanism that appropriate doping enhances mechanical performance while excessive doping leads to performance degradation.

### 3.3. Effect of SiO_2_ Concentration on the Friction Properties

**Figure 9:**
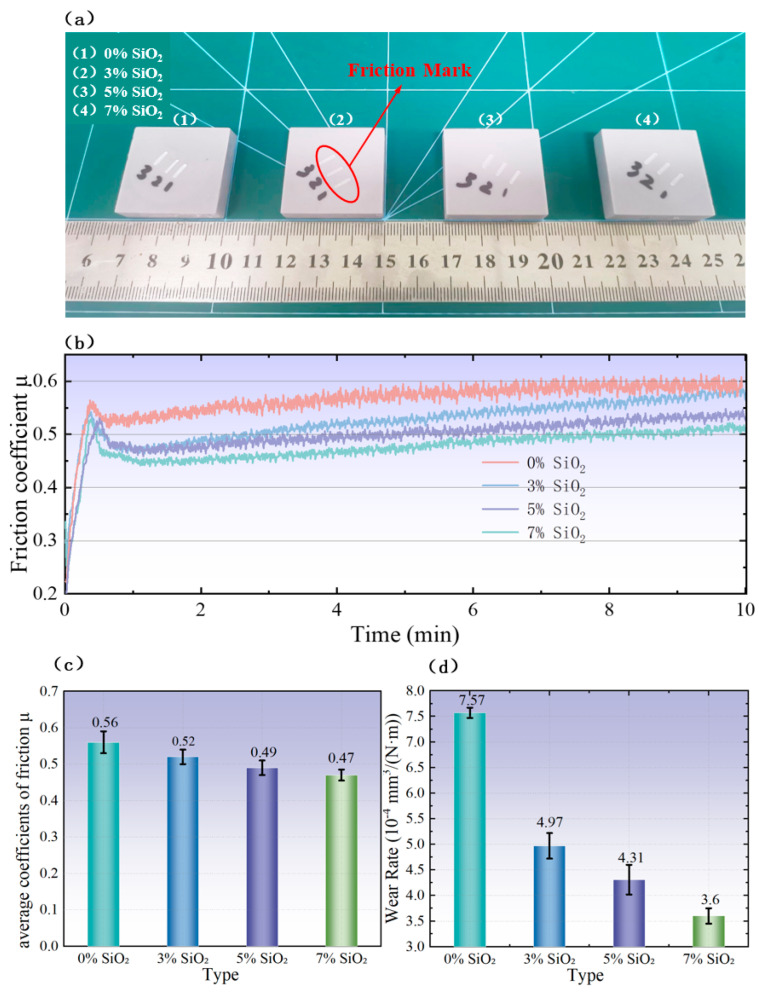
Friction experiment and results of photopolymer resin blocks with different SiO_2_ concentration gradients: (**a**) photopolymer resin blocks with different SiO_2_ contents (after friction test); (**b**) friction coefficient curves as a function of experimental time; (**c**) average friction coefficient under different SiO_2_ concentration gradients; (**d**) average wear rate under different SiO_2_ concentration gradients.

To investigate the influence of SiO_2_ powder addition on the friction properties of photopolymer resin, photopolymer resin blocks (30.3 mm × 30.3 mm × 9 mm in size) with four different SiO_2_ concentration gradients (0%, 3%, 5%, and 7% by weight) were fabricated via 3D printing, as illustrated in Figure 9a. Comparative surface wear scar experiments were subsequently conducted to evaluate the wear behavior of these resin blocks.

Friction tests were performed using the comprehensive material surface property tester described in Section 2.3, where a constant normal load of 30 N, a reciprocating frequency of 2 Hz, and a single sliding stroke of 5 mm were applied to the resin surface for a duration of 10 min. To ensure the reliability of test results, three parallel scratch treatments were carried out on each resin block. The friction coefficient measurement results of the photopolymer resin blocks with different SiO_2_ contents are presented in Figure 9b. After systematic data processing, the average friction coefficient for each SiO_2_ concentration gradient was calculated, as shown in Figure 9c.

As collectively illustrated in Figure 9b,c, with the continuous increase in SiO_2_ content, the friction coefficient curves exhibited a significant downward trend, accompanied by a reduction in the average friction coefficient. This observation indicates that the incorporation of SiO_2_ powder effectively enhances the anti-friction performance of the photopolymer resin.

Subsequently, the three wear scars on each resin block (for all SiO_2_ concentration gradients) were measured using the probe-type material surface wear scar measuring instrument introduced in Section 2.2. For each wear scar, measurements were conducted along four line directions perpendicular to the wear scar’s length. After excluding extreme values and calculating the average, the average wear volume of each resin block (corresponding to the four SiO_2_ concentrations) was obtained. The average wear rate of the four resin materials was then calculated using Formula (3), and the results are presented in Figure 9d.

It can be clearly observed from Figure 9d that the wear rate of the resin blocks decreases significantly with the continuous increase in SiO_2_ content, indicating a gradual improvement in the wear resistance of the photopolymer resin. The underlying enhancement mechanism is attributed to the higher hardness of SiO_2_ particles compared to the photopolymer resin matrix: the SiO_2_ particles act as a reinforcing phase that can effectively resist wear during the friction process, thereby leading to a continuous decrease in the overall wear rate of the composite material with increasing SiO_2_ content.

### 3.4. Effects of Corrugated Period and Amplitude on the Compressive Performance

In the investigation of the influence of honeycomb corrugation geometric parameters on the mechanical performance of symmetric corrugated hierarchical honeycombs (SCHH), a critical consideration is that the cylindrical support structures are inscribed within the honeycomb corrugations. Consequently, any variation in the geometric parameters of the honeycomb corrugations would inevitably induce corresponding changes in the parameters of the cylindrical structures. To strictly adhere to the single-variable principle and ensure the reliability of the experimental results, the cylindrical support structures described in Section 3.1 were removed for this specific phase of the study.

**Figure 10:**
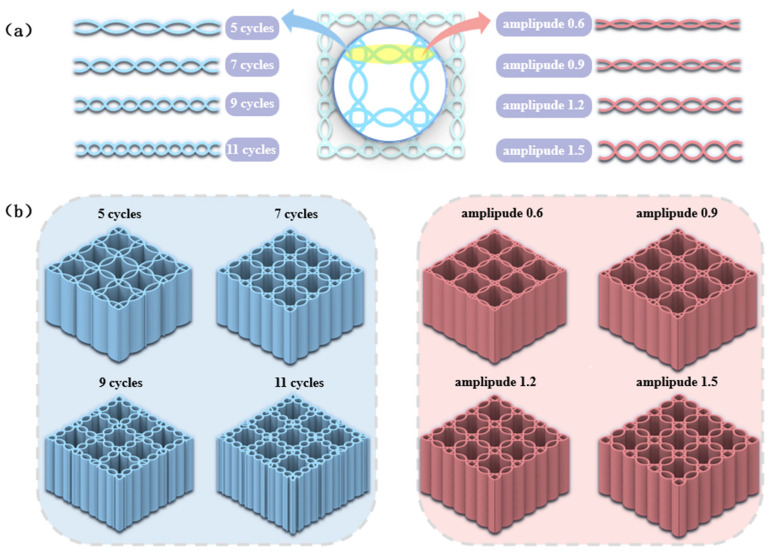
(**a**) Honeycomb corrugations with different periods and amplitude coefficients; (**b**) SCHH with different periods and amplitude coefficients.

To investigate the effects of the period and amplitude parameters of the honeycomb corrugated structure on the mechanical properties of SCHH, the total length, total width, and height of the honeycomb were kept constant. As shown in Figure 10a, four groups of symmetric corrugations were designed with 5 cycles, 7 cycles, 9 cycles, and 11 cycles, corresponding to corrugation periods of 5, 7, 9, and 11, respectively. Meanwhile, another four groups of symmetric corrugations were designed with amplitudes of 0.6 mm, 0.9 mm, 1.2 mm, and 1.5 mm (denoted as amplitude 0.6, amplitude 0.9, amplitude 1.2, and amplitude 1.5), corresponding to $h_{c}$ = 0.6 mm, 0.9 mm, 1.2 mm, and 1.5 mm, respectively. The honeycomb corrugations were arranged periodically to obtain the experimental models as illustrated in Figure 10b. Three parallel specimens were prepared for each group, followed by mechanical tests on the compressive properties of these models.

**Figure 11:**
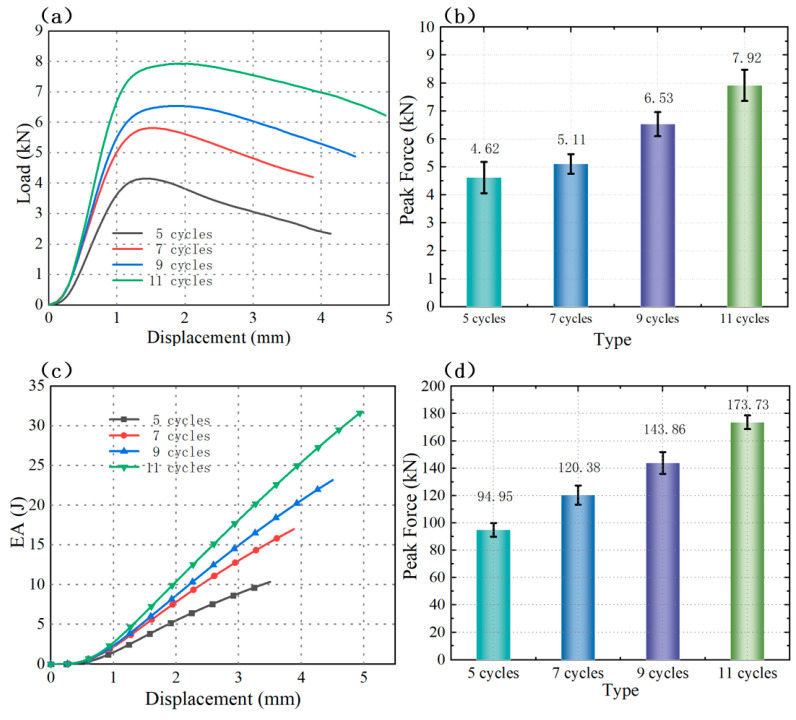
Mechanical performance comparisons of SCHH with different corrugation period numbers: (**a**) load–displacement curves; (**b**) peak load comparison; (**c**) energy absorption performance comparison; (**d**) compressive modulus comparison.

As clearly illustrated in Figure 11a,b, among the four corrugation period groups investigated, the specimen with 5 cycles exhibited the lowest load-bearing capacity, while the specimen with 11 cycles showed the highest load-bearing capacity. A distinct positive correlation was observed between the number of corrugation periods and the load-bearing capacity of the SCHH specimens: the greater the number of corrugation periods, the stronger the load-bearing capacity of the specimen. The underlying mechanism for this phenomenon can be elaborated as follows: with the increase in the number of corrugation periods, the effective surface area of the honeycomb corrugations increases significantly. This enlarged surface area enables more efficient dispersion of external compressive forces during the loading process, thereby reducing local stress concentrations and contributing to the sequential enhancement of both load-bearing capacity and peak load with increasing corrugation period number.

This positive correlation between corrugation period number and structural performance is further validated by the energy absorption and compressive modulus results. Figure 11c presents the energy absorption–displacement curves for different corrugation period numbers, which clearly demonstrate that the energy absorption capacity of the SCHH specimens increases sequentially as the number of corrugation periods increases. Additionally, Figure 11d shows the compressive modulus of the specimens under different period conditions, from which it can be observed that the compressive modulus also increases with an increasing number of corrugation periods. Collectively, these results confirm that increasing the corrugation period number can comprehensively enhance the mechanical performance of the SCHH structure.

**Figure 12:**
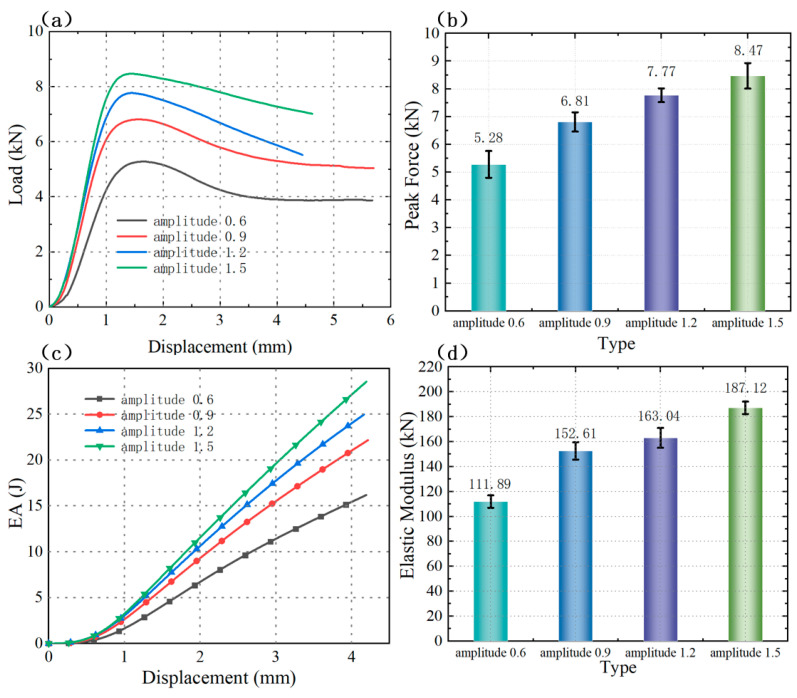
Mechanical performance comparisons of SCHH with different corrugation amplitudes: (**a**) load–displacement curves; (**b**) peak load comparison; (**c**) energy absorption performance comparison; (**d**) compressive modulus comparison.

As clearly illustrated in Figure 12a,b, among the four corrugation amplitude groups tested, the specimen with a 0.6 mm amplitude exhibited the lowest load-bearing capacity, whereas the specimen with a 1.5 mm amplitude demonstrated the highest load-bearing capacity. A distinct positive correlation was identified between corrugation amplitude and the load-bearing capacity of the SCHH specimens: the larger the corrugation amplitude, the stronger the load-bearing capacity of the specimen.

The underlying mechanism governing this trend is consistent with that observed for the corrugation period number: as the corrugation amplitude increases, the effective surface area of the honeycomb corrugations is significantly enlarged. This expanded surface area facilitates more efficient dispersion of external compressive forces during the loading process, thereby mitigating local stress concentrations and leading to the sequential enhancement of both load-bearing capacity and peak load with increasing corrugation amplitude.

This positive correlation between corrugation amplitude and overall structural performance is further corroborated by the energy absorption and compressive modulus results. Figure 12c presents the energy absorption–displacement curves for the different corrugation amplitude groups, which clearly indicate that the energy absorption capacity of the SCHH specimens increases sequentially as the corrugation amplitude increases. Additionally, Figure 12d displays the compressive modulus of the specimens under varying amplitude conditions, from which it can be observed that the compressive modulus also increases with increasing corrugation amplitude. Collectively, these findings confirm that increasing the corrugation amplitude can comprehensively improve the mechanical performance of the SCHH structure.

## 4. Discussion

In this study, 3D printing technology was employed to fabricate symmetric corrugated hierarchical honeycombs (SCHHs), their reinforced variant with cylindrical support structures (SCHH-AC), and resin-based honeycomb specimens modified with varying concentrations of SiO_2_. To systematically explore the factors governing the mechanical properties of these honeycomb structures, a comprehensive research approach was adopted, integrating quasi-static compression tests, friction and wear tests, and ABAQUS finite element simulation. The main conclusions drawn from this study are as follows:

Among all fabricated honeycomb configurations, the SCHH-AC specimen with 7 wt.% SiO_2_ doping exhibits the best overall compressive performance. Compared with the pure resin SCHH structure and other SiO_2_-modified SCHH-AC specimens, this sample possesses the highest peak load, markedly improved energy absorption capacity, and an apparently higher compressive modulus. This finding is consistent with the conclusion reported by Xia and Meng et al. [45] that KH550/KH570 modified nano-SiO_2_ can effectively enhance the compressive strength and stiffness of epoxy resin composites. Similarly, Roopavath et al. [46] introduced nano-SiO_2_ into alginate–gelatin hydrogel scaffolds and achieved an obvious improvement in compressive modulus, which is in good agreement with the present experimental results. Jeong et al. [47] developed photocurable elastomers incorporated with crosslinkable SiO_2_ nanofillers, increasing the tensile strength and hardness of 3D-printed structures by 87% and 52%, respectively. Nevertheless, their research focused mainly on tensile and resilience properties, whereas this study concentrates on the combined performance of compressive strength, energy absorption capacity, and compressive modulus.

When the SiO_2_ mass fraction increases to 9 wt.%, severe particle agglomeration occurs within the resin matrix, damaging the structural integrity of the matrix as well as the interfacial bonding between nanoparticles and resin. Consequently, the mechanical performance of the honeycomb structure deteriorates significantly, with pronounced reductions in peak load, energy absorption capacity, and compressive modulus compared with the optimal 7 wt.% doping case. Guo et al. [48] also found that single-sized nano-SiO_2_ filled in epoxy resin tends to form agglomerates, sharply increasing the resin viscosity and imposing an adverse effect on thermomechanical properties. The novelty of this work lies in systematically adjusting the SiO_2_ doping concentration from 0 wt.% to 9 wt.%, which quantitatively reveals the direct correlation between particle agglomeration and mechanical performance degradation.

The incorporation of nano-SiO_2_ into the resin matrix exerts a remarkable positive regulation on the friction and wear performance of honeycomb structures. Existing literature has verified the improvement effect of SiO_2_ on tribological behavior in various composite systems. Zhang et al. [49] reported that copper-based metal–organic frameworks combined with nano-SiO_2_ produce a synergistic effect in carbon fiber-reinforced epoxy composites, transforming the wear mechanism from abrasive wear to adhesive wear and reducing the specific wear rate to 5.64 × 10^−7^ mm^3^/(N·m). Zhang et al. [50] adopted Cu-coated SiO_2_ to optimize the interfacial bonding between particles and matrix in copper-based brake pads, restraining the shedding of SiO_2_ and facilitating the formation of a stable friction platform. Sugozu et al. [51] systematically compared the effects of nano- and micro-sized SiO_2_ on polymer friction materials and confirmed that uniformly dispersed nano-SiO_2_ can effectively stabilize the friction coefficient and reduce the specific wear rate. However, the above studies rarely involved the influence of SiO_2_ doping on the friction coefficient and wear rate of resin-based materials, which was comprehensively investigated in the present work. In addition, geometric parameter analysis indicates that increasing the wave period and wave amplitude of symmetric corrugated hierarchical honeycombs can further enhance the compressive strength and energy absorption capacity of the structure.

Future research can optimize the dimensions, arrangement of cylindrical supports, and topological configuration of corrugated honeycombs to further tap the mechanical potential of symmetric corrugated hierarchical honeycombs. Additionally, studies on multiphase nanofiller composite modification, particle surface modification, and interface regulation are needed to suppress nano-SiO_2_ agglomeration and enhance composite performance.

In addition, the resin-based system can be extended to metals, ceramics, and carbon fiber-reinforced composites, and cross-scale fabrication research combined with different 3D printing processes can provide comprehensive theoretical and technical support for the engineering application of such hierarchical honeycombs in high-end equipment and extreme environments.

## Figures and Tables

**Table 1:** Geometric parameters of the SCHH-AC.

Parameter	l (mm)	h (mm)	w (mm)	$t_{c}\;(mm)$	$t_{a}\;(mm)$
value	30.3	18	30.3	0.53	0.53

**Table 2:** The main parameters of the 3D printer.

Parameter	Pixel Accuracy (μm)	Optical Resolution	Layer Thickness (mm)	Curing Time (min)
value	65	3840 × 2160	0.1	15

**Table 3:** Material parameters of resin.

Type	Density (g/cm^3^)	Young’s Modulus (MPa)	Poisson’s Ratio
0% SiO_2_ resin	1.20	462.058 ± 0.861	0.354 ± 0.003
5% SiO_2_ resin	1.26	469.250 ± 0.518	0.346 ± 0.004

**Table 4:** Number of nodes and elements.

Mesh Size (mm)	Number of Nodes	Number of Elements
0.50	78,847	48,060
0.55	65,348	40,524
0.60	47,120	28,560

## Data Availability

The original contributions presented in this study are included in the article. Further inquiries can be directed to the corresponding authors.

## Abbreviations

The following abbreviations are used in this manuscript:

SCHH	Symmetric corrugated hierarchical honeycombs
SCHH-AC	Symmetric corrugated hierarchical honeycomb with embedded cylindrical supports
SCHH-AC-5%Si	Symmetric corrugated hierarchical honeycomb with embedded cylindrical supports containing 5 wt.% SiO_2_
